# Feasibility of Low-Sodium, High-Potassium Processed Foods and Their Effect on Blood Pressure in Free-Living Japanese Men: A Randomized, Double-Blind Controlled Trial

**DOI:** 10.3390/nu13103497

**Published:** 2021-10-03

**Authors:** Yoko Umeki, Hitomi Hayabuchi, Hisashi Adachi, Masanori Ohta

**Affiliations:** 1 Graduate School of Health and Environmental Sciences, Fukuoka Women’s University, Fukuoka 813-8529, Japan; umeki@fwu.ac.jp (Y.U.); h.hayabuchi@cc.nara-wu.ac.jp (H.H.); 2 Department of Food Science and Nutrition, Faculty of Human Life and Environment, Nara Women’s University, Nara 630-8506, Japan; 3 Department of Community Medicine, Kurume University School of Medicine, Kurume-shi 830-0011, Japan; hadac@med.kurume-u.ac.jp

**Keywords:** sodium, potassium, urinary Na/K ratio, blood pressure, hypertension, free-living settings, double-blind, randomized controlled trial, food environment improvement

## Abstract

We aimed to verify the effect of new low-sodium high-potassium seasonings and processed foods containing poly-γ-glutamic acid on blood pressure in free-living settings. To this end, we conducted a randomized, double-blind controlled trial on 187 Japanese men, aged 35–67 years, who did not use antihypertensives. Participants were randomly allocated to an intervention (*n* = 93) or a control group (*n* = 94). They were given a boxed lunch and miso soup (average Na and K content for the intervention group: 1175 and 1476 mg; for the control group: 2243 and 703 mg, respectively). Blood pressure was measured three times every morning for 1 week immediately before and during the final week of the trial. On the day before and the final day of the intervention period, 24 h urine samples were collected. After intervention, the intervention group showed a significantly stronger decrease in the urinary sodium-to-potassium ratio than the control group (*p* < 0.001). The mean difference in systolic blood pressure change after adjustment for baseline values between the two groups was −2.1 (95% CI: −3.6, −0.6) mmHg. Compliance between the groups was similar, suggesting successful blinding. In conclusion, the use of new seasonings and processed foods aimed at lowering blood pressure in free-living settings may be feasible and effective.

## 1. Introduction

Hypertension is one of the most important risk factors for cardiovascular disease [1,2,3,4,5], and substantial efforts to prevent and control it have been made over recent decades. However, the prevalence of hypertension in Japan has increased with age, exceeding 50% in men over the age of 50, and is likely to increase further as the population ages [6].

The importance of hypertension management warrants not only pharmacotherapy but also non-pharmacological treatment. In Japan in 2012, the “National Health Promotion Movement for the 21st Century” (Health Japan 21 (the second term)) [7,8] set a target to, by 2022, reduce systolic blood pressure (SBP) by 2.3 mmHg as a public health measure for hypertension. To lower blood pressure (BP), objectives were set to reduce salt intake (<8 g/d), increase fruit and vegetable intake (>350 g/d), and reduce the number of obese people through nutritional and dietary measures. To achieve these goals, it is necessary to improve the food environment by spreading awareness of salt reduction through mass media, improving school lunch and food service industry menus, and reducing the amount of salt used by the food industry in seasonings and processed foods.

Several previous studies have shown that high salt intake increases BP whereas lowering salt intake decreases it [1,2,3,4,5,9,10,11,12,13,14,15,16,17,18,19,20,21]. It has been reported in a cross-sectional analysis that the sodium-to-potassium ratio (Na/K ratio) is associated with BP [22,23,24,25]. In addition, reduction in the Na/K in hypertension have been associated with a decrease in SBP [26]. High potassium (K) intake also inhibits sodium (Na) reabsorption, which tends to lower BP [27,28]. Several intervention studies have examined the lowering of BP using salt substitutes with a low Na and high K (mainly KCl) content [18,20]. KCl has a salty taste as an inorganic salt; however, a bitter taste is simultaneously perceived [29]. When added as a salt substitute for NaCl, the bitter taste often impacts the flavor of the seasoning, preventing the addition of a sufficient amount of KCl.

Recently, low-Na, high-K seasonings and processed foods with a small amount of added poly-γ-glutamic acid (PGA), to mask the bitter taste of K, have been developed. PGA, a polymer of glutamic acid which is formed by bacterial fermentation, is also the sticky ingredient in natto, a traditional Japanese food. Meals prepared using these seasonings and processed foods cannot be distinguished from regular meals in either appearance or taste [30,31]. We speculated that these seasonings may be useful in intervention studies aimed at determining the impact of a low-Na and high-K diet on BP.

In the present study, we conducted a randomized controlled trial using boxed lunches and miso soup flavored using these new techniques as intervention meals. The aim of this study was to verify the feasibility of interventions using these new seasonings in free-living settings and to evaluate the effect of a low-Na, high-K diet on BP in Japanese men.

## 2. Materials and Methods

### 2.1. Study Design and Participants

**Figure 1:**
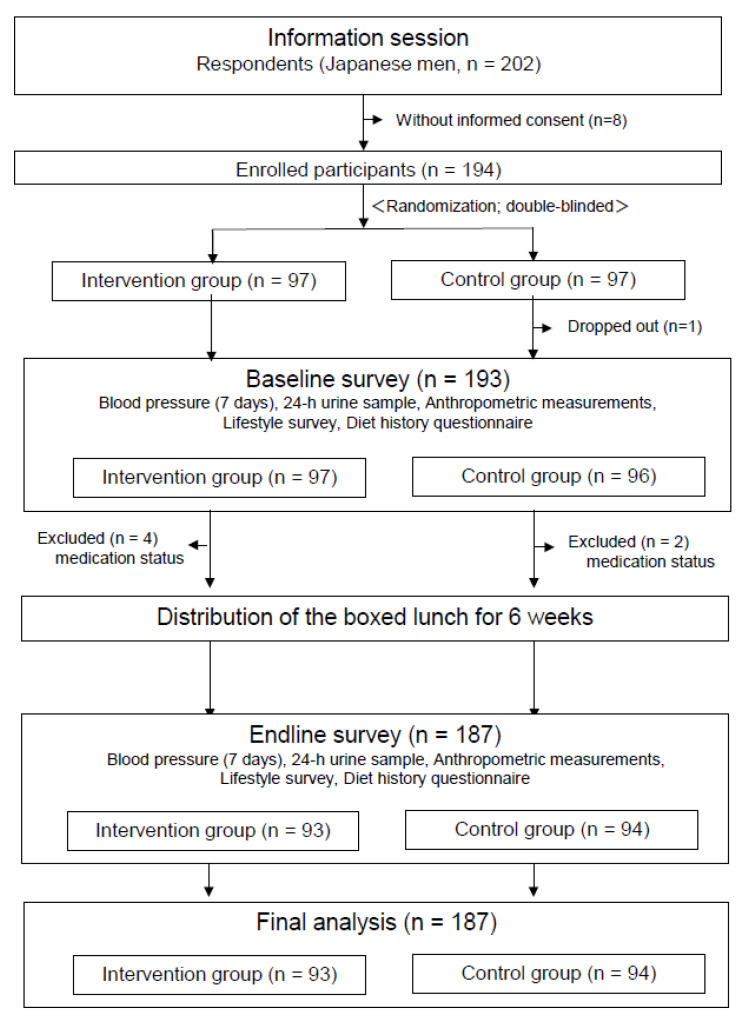
Flowchart of the research process.

The sample size necessary to estimate the effect of salt reduction on BP lowering by 4.6 ± 2.1 mmHg [32] (alpha = 0.05 and power = 0.08) was 133. An additional 30% was added to compensate for eventual losses and unknown-confounding factors. We finally made a plan to recruit approximately 200 men working in the city hall and a private company in Omuta City, Fukuoka, Japan. Included participants agreed with the purpose of the study, were men aged ≥ 35 years, and had SBP ≥ 130 mmHg or diastolic BP (DBP) ≥ 85 mmHg at the regular health check-ups that year. Those who were taking antihypertensive drugs, and patients with renal disease were excluded. A total of 202 men aged 35–67 years attended the orientations, among whom 194 provided written informed consent (consent rate: 96.0%) and were enrolled as participants. After these 194 male participants were registered, they were randomly allocated to either the intervention or control group (97 participants each; Figure 1). Confirmation of participation requirements and allocation to different groups were conducted by the medical information department at the participant’s workplace. Participants were assigned to two groups using cluster-randomized allocation by workplace and age. A random number table was used to generate the order table for randomized allocation. A staff member of the medical information department at the participant’s workplace, who was not directly involved with the research staff, managed the randomization order table and performed the allocation.

### 2.2. New Low-Na, High-K Processed Foods

New low-Na, high-K processed foods were used in the preparation of the meals, in the form of boxed lunches and miso soup, provided to the intervention group. The nutritional value of the major low-Na, high-K seasonings and processed foods used in the intervention study are shown in the Appendix A (Table A1).

### 2.3. Intervention Meals: Boxed Lunches and Instant Miso Soup

We used boxed lunches and cups of miso soup as the intervention meals. The lunches were manufactured by a food manufacturing factory located nearby the study locale and delivered to the work sites daily during the intervention period. Boxed lunches were prepared from 10 different menus, consisting of rice, fish and meat side dish, such as salted salmon or teriyaki chicken, and a vegetable side dish, such as pickled cucumber in soy sauce or pickled radish. Both the low-Na instant miso soup and the regular instant miso soup were produced by Ichibiki Co., Ltd. (Nagoya, Japan). The intervention and control group received the same menu on the same day; all meals consisted of the same ingredients and processed foods with the same appearance, differing only in the use of the new low-Na, high-K processed foods versus the regular versions. We asked the male participants to prepare the instant miso soup by diluting it with hot water. To standardize the concentration and amount of miso soup, we provided soup bowls marked with the amount of hot water to pour and attached instructions on how to prepare the soup. Moreover, we requested all male participants to, after each lunch, record how much of each boxed lunch and miso soup they consumed.

Table 1 presents the calculated nutritional values of the boxed lunches. The energy content, and specific nutritional content of the boxed lunches and instant miso soup provided to the intervention and control group were calculated using the values in the Standard Tables of Food Composition in Japan 2005 (Fifth Revised and Enlarged Edition), while analytical values were used to calculate those of the new low-Na, high-K processed foods. The average energy, protein, fat, and carbohydrate content of the boxed lunches (average of 10 samples) and instant miso soup (average of 10 samples) did not significantly differ between the intervention and control groups. The average Na and K content per boxed lunch for the intervention group was 783 ± 135 mg (mean ± SD) and 1156 ± 199 mg (0.7 ± 0.1 of Na/K ratio), respectively, whereas that for the control group was 1654 ± 220 mg and 635 ± 142 mg (2.7 ± 0.8 of Na/K ratio), respectively—these values significantly differed between the two groups (*p* < 0.001 for all). The average Na and K content of the instant miso soup per serving (cup) was 392 ± 17 mg and 320 ± 38 mg (1.2 ± 0.2 of Na/K) for the intervention group, respectively, and 589 ± 20 mg and 68 ± 21 mg (9.7 ± 3.7 of Na/K) for the control group, respectively—these values also significantly differed between the two groups (*p* < 0.001 for all).

### 2.4. Intervention Schedule

The intervention was performed using a randomized parallel design over 6 weeks, from the end of June to the beginning of August 2010. For the double-blind study, neither participant, nor boxed lunch distributor, or the researcher was aware of the composition of the meals, except for the person responsible for producing boxed lunches. BP was measured three times every morning for one week immediately before the intervention started. The male participants answered the self-administered diet history questionnaire (DHQ), which included several questions regarding lifestyle risk factors, and 24 h urine samples were collected on the day prior to the start of the intervention. Furthermore, the height and body weight of the male participants were measured by research staff on the first day of the intervention. During the intervention period, the male participants in the intervention group received a low-Na, high-K boxed lunch and miso soup as their work day lunch (5 days per week), while those in the control group received a regular boxed lunch and miso soup. The male participants were requested to consume the boxed lunch and a cup of miso soup. Prior to the intervention period, the male participants were asked not to change their regular diet during the intervention study, except for lunch. The male participants kept a rough dietary record on how much of each boxed lunch they consumed.

BP was measured again during the last week of the intervention period. The male participants also answered the DHQ again during this period. The second 24 h urine sample collection was performed on the final day of the intervention, while height and body weight were measured again on the day after the intervention was completed. The completed DHQ, dietary records, and other questionnaires were revised by trained staff. After the intervention was completed, participants were questioned which diet do you think you have consumed in this study, the low-Na and high-K or the regular one.

### 2.5. Blood Pressure Measurement

An automated sphygmomanometer (blood pressure monitor HEM-7080IC; Omron Healthcare Co., Ltd., Kyoto, Japan) was provided to all male participants. Participants measured their BP at home three times every morning immediately after getting out of bed for 7 consecutive days. We collected the sphygmomanometers from the participants after the intervention trial to download the measurement data. In cases when measurements were made in the afternoon and when measurement frequency was more than four times, the data were excluded. As we have previously reported, the adherence rate regarding the frequency and time of BP measurement was high, approximately 90% [33]. BP values were extracted directly from the electronic records of the sphygmomanometers. The mean BP was calculated at baseline and after the intervention as an individual representative value for further analysis.

### 2.6. Diet History Questionnaire

The dietary habits of the male participants over the preceding month were assessed using a previously validated DHQ [34,35]. The DHQ comprised a 22 page structured food frequency questionnaire consisting of more than 400 questions, from which the dietary intake of 151 foods and over 50 nutrients was calculated. Items and portion sizes were derived primarily using data from the National Nutrition Survey of Japan and several recipe books for Japanese dishes [34]. The DHQ has been tested for validity using several methods and has been used in several studies conducted in Japan [36,37,38]. The DHQ did not take into account the differences in nutritional composition between the boxed lunches and miso soup for the intervention and control group.

### 2.7. Urine Collection

A single 24 h urine sample was collected at baseline, and on the day after the intervention was completed. Post-intervention urine storage was conducted over the last day of the study, as was done on the day the boxed lunch was consumed. The 24 h collection method has been described in detail elsewhere [39]. The male participants were asked to collect all specimens in the morning after they had discarded their first urine, and to collect their last specimen the next morning at the same time when they had discarded their first urine sample the previous morning. The times when the urine collection was started and finished were recorded. Participants who forgot or failed to collect urine were asked to record an estimated volume, which was added to the actual volume of urine collected. The time of urine collection was then adjusted based on the urine collection record, and the total volume was calculated. The samples of collected urine were sent to the Fukuoka Institute of Occupational Health, Japan, and analyzed for urinary Na and K concentrations using ion-selective electrodes. Creatinine levels were analyzed using enzymatic methods. After urine samples were analyzed, urine containing creatinine excretion in the range of 14.4–33.6 mg/kg (male reference) was accepted [40]. This criterion was used for participant inclusion. The Na-to-K excretion ratio was calculated according to the Intersalt Cooperative Group finding that this variable is more strongly associated with an antihypertensive effect than the excretion of Na or K alone [41].

### 2.8. Anthropological Measurements and Other Variables

Height and body weight were measured by trained staff using a body composition analyzer (TBF-210, Tanita Co., Ltd., Tokyo, Japan). Body mass index (BMI) was calculated as body weight divided by the square of the height (kg/m^2^). Habitual physical activity was assessed as the length of time performing certain types of physical activity per week (intermediate activities, sports activities, and muscle training). At the baseline and endline surveys, participants were asked to self-report their physical activity, medication, and smoking status using self-administered questionnaires, which were checked by research staff at each site.

### 2.9. Statistical Analyses

Figure 1 shows the chart flow of male participants selection and their allocation to different groups. One participant in the control group dropped out owing to hospital admission during the intervention period. During the intervention period, six participants (four in the intervention group and two in the control group) were excluded because they were using antihypertensive medication or other drugs that strongly affected the urinary excretion of Na or K. Ultimately, 187 participants (93 in the intervention group and 94 in the control group) were included in the analyses.

Statistical analyses were performed using JMP Pro statistical software (version 15.0; SAS Institute Inc., Cary, NC, USA). Mean values (standard deviation (SD) or proportion (%)) were calculated for the baseline characteristics. Mean values of numerical variables between the intervention and control group were compared using the *t*-test, while proportions of categorical variables were calculated using the chi-square test (Fisher’s exact test was used whenever the expected cell frequencies were ≤5).

The effect of the intervention on BP and laboratory test results of urine collection (Na, K, and urinary Na/K ratio) was analyzed on an intention-to-treat basis. Differences in BP change between the groups during the intervention period were compared using analysis of covariance (ANCOVA) with adjustment for baseline BP. Three models were used: Model 1 was adjusted for baseline BP only; Model 2 was adjusted for baseline BP, age, smoking status, change in body weight, and changes in physical activity [1,42]; and Model 3 was adjusted for the abovementioned variables as well as for dietary risk factors (changes in the intake of alcohol [43,44,45,46], omega-3 polyunsaturated fat [47,48,49], energy [50,51], dietary fiber [52], calcium [53,54,55], magnesium [56], and protein [57,58,59,60]). Two-sided *p*-values < 0.05 were considered statistically significant in all analyses.

## 3. Results

### 3.1. Participant Characteristics

Table 2 presents the male participant baseline characteristics. For both groups, no significant differences in age, height, body weight, BMI, SBP, or DBP were found regarding the proportion of smokers, participants with obesity, and those with a history of hyperuricemia, hyperlipidemia, or diabetes (all *p* > 0.05). According to the diagnostic criteria for hypertension in home BP (SBP ≥135 or DBP ≥85), 79 participants (41 in the intervention group and 38 in the control group) had hypertension at baseline, with no difference in the proportion of hypertensive participants between the two groups. The majority of the participants in both groups were desk workers. The intake of selected nutrients or food groups evaluated using DHQ were no significant differences between the two groups except for fruits intake. In addition, there was no significant difference in physical activity and urinary Na and K excretion between the two groups.

### 3.2. Compliance and Blinding

The proportion of male participants who had consumed the provided boxed lunches in the intervention (92.2%) and control group (91.0%) was the same (*p* = 0.44). Further, 73.1% of participants in the intervention group and 68.4% in the control group consumed the miso soup, showing no significant difference between the groups (*p* = 0.31).

In a questionnaire administered after the intervention period, 55.9% of the participants in the intervention group and 59.6% in the control group reported that they thought they had consumed their regular diet throughout the intervention period (*p* = 0.61).

### 3.3. Changes in Dietary Intake and Lifestyle Factors

Table 3 presents the changes in the intake of selected nutrients assessed using the DHQ. The changes did not differ significantly between the groups for almost all nutrients apart for calcium (adjusted between-group difference was significant: *p* = 0.04). For physical activity, intermediate activities were increased in the intervention group compared to those in the control group (adjusted between-group difference: *p* = 0.049).

### 3.4. Changes in Urinary Excretion of Na and K

The changes in urinary excretion of Na and K are shown in Table 3. The difference in the change in Na excretion between the control and intervention group after adjustment for baseline values by ANCOVA was −156 mg/d (95% CI: −635, 324). Although not statistically significant, the results indicated that there was a larger decrease in Na excretion in the intervention group. Furthermore, K excretion increased by 435 mg/d (95% CI: 291, 577), and the urinary Na/K ratio decreased by −0.7 (95% CI: −0.9, −0.5) in the intervention group. In the control group, K excretion decreased by −258 mg/d (95% CI: −375, −140), while the urinary Na/K ratio increased by 0.3 (95% CI: −0.0, 0.5). The mean difference in change between the two groups after adjustment for baseline values was 646 mg/d (95% CI: 477, 815); in other words, the increase in K excretion was much larger in the intervention than in the control group. Similarly, the change in the urinary Na/K ratio was also significantly different between the groups (*p* < 0.001), with a larger decrease in the intervention than in the control group.

### 3.5. Changes in BP

Table 4 shows the changes in BP before and after the intervention in each group. The mean SBP did not change in the intervention group, but increased by 1.7 (95% CI: 0.8, 2.6) mmHg in the control group. The mean difference in change in SBP after adjustment for baseline values between the two groups was −2.1 (95% CI: −3.6, −0.6; Model 1) mmHg, −2.0 (95% CI: −3.6, −0.5; Model 2) mmHg, and −2.0 (95% CI: −3.6, −0.4; Model 3) mmHg, showing significant changes in SBP between the groups. For DBP, the mean difference in change was −0.9 (95% CI: −2.0, 0.2; Model 1) mmHg, −0.9 (95% CI: −2.0, 0.3; Model 2) mmHg, and −0.9 (95% CI: −2.1, 0.3; Model 3) mmHg, showing no significant difference between the groups.

## 4. Discussion

In this intervention study, low-Na, high-K boxed lunches and miso soup containing approximately 1000 mg less Na (2.7 g of NaCl) and 780 mg more K per serving, compared to the regular versions, were used. The present study is the first double-blind randomized controlled trial examining the effects of consuming low-Na, high-K seasonings and processed foods containing small amounts of PGA on blood pressure. The results showed that the intake of low-Na, high-K lunches enhanced urinary excretion of Na and suppressed the increase in SBP.

We would like to highlight one key point of this study: the intervention did not require male participants to avoid salty foods or increase their intake of K-rich foods. Intervention studies using a double-blind method to limit Na intake have generally been challenging because of the ease of knowing whether participants were assigned to the intervention or control group based on the taste (saltiness) of the food provided. Moreover, the unpleasant taste attributed to low-Na (tasteless) and high-K (bitter taste) seasonings may result in undesirable acceptability and low compliance [61,62,63,64], particularly in East Asian populations that generally prefer highly salted meals [65]. A more palatable low-Na diet is necessary to prevent hypertension. In the present intervention, the percentage of participants who consumed the provided boxed lunches and miso soup was the same in both the intervention and control groups. Therefore, the boxed lunches and miso soup prepared with low-Na, high-K seasonings and processed foods provided to the intervention group were comparable in taste to the regular versions. In other words, blinding in this interventional study was considered successful.

The intervention group showed a significant increase in urinary K excretion. Na excretion decreased in both groups and the difference between the groups after adjustment was not significant. The lack of difference in urinary Na excretion could have been due to the study period having included the summer season—it is possible that the results are unclear due to sweating. After the intervention, there was a decrease in urine volume in both groups, suggesting that the high-temperature environment caused water and a considerable amount of Na to be lost through perspiration. In addition, the intervention group consumed a large amount of K, which inhibits Na reabsorption, and may have resulted in the excretion of more Na in the urine [27,28]. This may have caused the similar urinary Na excretion values between the groups. Our results indicate that these new seasonings and processed foods with a low-Na, high-K content and a small amount of PGA may be feasible for use in intervention trials aimed at lowering BP in free-living settings.

Moreover, the present study showed that low-Na and high-K intake promotes urinary excretion of K and suppresses the increase in SBP. The previous study reported that reducing salt by 3 g per day was estimated to decrease SBP by 1.8–3.5 mmHg [66]. In the present study, the difference in adjusted blood pressure between the intervention group and the control group was 2.1 mmHg, with a salt reduction of 2.7 g per lunch meal. The urinary K excretion of the intervention group increased by 435 mg after the intervention, suggesting that they compensated for the insufficient K intake. K promotes urinary excretion of Na [27,28], suggesting that the use of low-Na and high-K foods in this study may prevent an increase in blood pressure. The significant decrease in the change in dietary calcium intake in the intervention group (Table 3) may have influenced the antihypertensive effect. The consumption of high K-containing boxed lunch in the intervention group may have further affected the antihypertensive effect, as it has been reported to increase urinary calcium excretion [67].

Randomized trials of reduced sodium and increased potassium intake have shown a clear BP lowering effect when using this substitute. In particular, the relationship between potassium intake and BP has been reported in meta-analyses of randomized controlled trials that lasted ≥4 weeks [68]. Most of the trials adopted were in adults with hypertension, and the effect of potassium supplementation on lowering BP was greater in hypertensive patients and those with higher sodium intake. The results showed a U-shaped relationship between BP and potassium excretion, with increased potassium excretion and increased BP in hypertensive patients treated with antihypertensive drugs, but not in untreated hypertensive patients. The meta-analysis conducted by The Evidence-based Practice Center of Agency for Healthcare Research and Quality in the U.S. reported that the use of potassium-enriched salt substitutes instead of sodium lowered SBP and DBP by an average of 5.58 mmHg and 2.88 mmHg, respectively [69]. A meta-analysis conducted by Peng et al. reported that the effect of salt substitutes on BP was greater in hypertensive patients. However, the effect was smaller in individuals who were not hypertensive, but the results may not have been clear due to the small number of trials involving people without hypertension [70].

In the present study, the effect of intervention with low-Na and high-K foods on SBP was approximately −2.0 mmHg, which was smaller than that shown in these meta-analyses. The present study population consisted of 79 participants (42.2%) who had hypertension in their home BP at baseline. However, none of them were being treated for hypertension because those taking blood pressure lowering medication at baseline were excluded from the study.

The average urinary Na excretion in all participants was 4906 mg, which was higher than the average Na intake in people in their 40s according to the 2010 National Health and Nutrition Examination Survey, but almost the same as that estimated from the urinary Na excretion in INTERMAP. In general, dietary record method surveys such as the National Health and Nutrition Examination Survey are known to have a limitation of reporting error, which is likely to differ from the results of the present study. Therefore, the dietary salt intake in the participants is considered to be similar to the general intake of Japanese people. The results of this study suggest that a low-sodium, high-potassium diet significantly lowers BP in Japanese men in general, including hypertensive patients not taking medication.

Several studies [71,72] have suggested that salt reduction may have greater BP-lowering effects in women, older individuals, and individuals with higher SBP (hypertensive patients). The male participants in the present study were relatively young (mean age: 48.1 ± 7.3 years) and had a relatively low BP (SBP: 128.0 ± 14.2 mmHg; DBP: 83.1 ± 10.2 mmHg). On these bases, interventions using the same seasonings and processed foods may be effective in other populations and may be further improved by more radical intervention (e.g., preparing all meals using these seasonings).

This study had a few limitations. First, 24 h urine samples were collected only once at baseline and once after the intervention period. Day-to-day variation in the urinary excretion of Na and K makes it difficult to observe their true habitual intake [73]. Second, an unexpected increase in BP was observed in the control group, and we could not identify any clear reason for such increase. Third, regarding the change in dietary habits due to the lunch intervention: although we were able to confirm that there was no change in habitual dietary intake before and after the intervention using a DHQ, it is possible that the meal form of the lunches served were different (e.g., before the intervention, lunches were eaten at restaurants or cafeterias instead of boxed lunches). Since both groups were served lunch under the same conditions, this was not expected to affect the results regarding differences between the two groups, but it may have influenced the changes before and after the intervention. The change in urinary Na and K excretion in the control group suggests that their eating habits have changed from before the intervention. The results in Table 3 indicate that the significant decrease in urinary sodium excretion in both groups may be resulted in urine volume. The significant increase in SBP in the control group as shown in Table 4 could be due to the decrease in potassium excretion shown in Table 3. Fourth, in this study, BP values were evaluated by three measurements within one hour after waking, after urination, and before breakfast. According to the previous studies of home BP in Japanese subjects, it has been reported that BP values were higher in the morning than in the evening [74]. Therefore, we are not clear whether the results of this study will be the same if BP was evaluated using the average of two measurements morning and evening each, which is the method of measuring home BP recommended by the Japanese Society of Hypertension [75]. Finally, our study participants were recruited from a single regional city and all participants were adult male office workers. Therefore, the generalizability of the results is limited. Further research to establish appropriate usage of these seasonings to lower BP among a larger sample is required, as well as in other populations such as women and individuals in other age groups.

## 5. Conclusions

Using new low-Na, high-K seasonings and processed foods containing a small amount of PGA, we performed a randomized, double-blind controlled trial in a free-living setting. We found that a high K intake promoted urinary excretion of Na and suppressed the increase in BP.

## Figures and Tables

**Table 1:** Nutritional content of boxed lunches and miso soup.

	Intervention Group		Control Group		*p*-Value *
	Mean	SD	Mean	SD	
Boxed lunches ^a^					
Energy (kcal)	724	32	740	40	0.348
Protein (g)	28.1	2.7	28.8	2.8	0.596
Fat (g)	20.9	4.5	21.1	4.8	0.928
Carbohydrates (g)	98.1	7.0	101.7	9.0	0.323
Sodium (mg)	783	135	1654	220	<0.0001
Potassium (mg)	1156	199	635	142	<0.0001
Sodium-to-potassium ratio	0.7	0.1	2.7	0.8	<0.0001
Instant miso soup ^b^					
Energy (kcal)	20	5	23	3	0.140
Protein (g)	1.6	0.2	1.7	0.3	0.411
Fat (g)	0.6	0.2	0.8	0.2	0.054
Carbohydrates (g)	2.2	0.5	2.3	0.2	0.587
Sodium (mg)	392	17	589	20	<0.0001
Potassium (mg)	320	38	68	21	<0.0001
Sodium-to-potassium ratio	1.2	0.2	9.7	3.7	<0.0001

Values are presented as mean ± standard deviation (SD). The nutritional value of general foods was determined using the values in the Standard Tables of Food Composition in Japan 2005 (Fifth Revised and Enlarged Edition), while that of the new low-Na, high-K processed foods were determined using analytical values. ^a^ Average nutritional content of the 10 boxed lunches used in the study ^b^ Average nutritional content of the 10 miso soups used in the study. * *p*-value for comparison of the nutritional value of boxed lunches and miso soup between the intervention and control group calculated using the *t*-test.

**Table 2:** Baseline characteristics of the study male participants.

	Intervention Group (*n* = 93)		Control Group (*n* = 94)		*p*-Value *
	Mean	SD	Mean	SD	
Age (years)	48.1	7.4	48.0	7.2	0.936 ^c^
Physiological data					
Height (cm)	171.1	6.2	171.4	5.5	0.725 ^c^
Weight (kg)	71.1	9.3	72.7	11.1	0.303 ^c^
BMI (kg/m^2^) ^a^	24.3	3.0	24.7	3.1	0.399 ^c^
SBP (mmHg)	128.7	15.7	127.3	12.6	0.485 ^c^
DBP (mmHg)	83.5	10.4	82.7	10.1	0.617 ^c^
Smoking status					0.189 ^d^
Current smoker	38	(40.9)	28	(29.8)	
Past smoker	21	(22.5)	20	(21.3)	
Non-smoker	34	(36.6)	46	(48.9)	
Overweight status ^a^					0.833 ^d^
Overweight	25	(26.9)	29	(30.9)	
Obese	6	(6.5)	6	(6.4)	
Presence of diseases					
Hyperuricemia	2	(2.2)	5	(5.3)	0.444 ^c^
Hyperlipidemia	2	(2.2)	0	(0.0)	0.246 ^c^
Diabetes	0	(0.0)	1	(1.1)	1000 ^d^
Self-reported dietary intake ^b^					
Energy (kcal/d)	2203	555	2224	630	0.811 ^c^
Protein (g/d)	65.8	18.8	67.9	22.7	0.484 ^c^
Fat (g/d)	58.9	22.3	64.0	33.5	0.236 ^c^
n-3 polyunsaturated fat (g/d)	2.50	0.98	2.86	1.75	0.086 ^c^
Carbohydrates (g/d)	279.8	87.3	277.5	69.4	0.841 ^c^
Dietary fiber (g/d)	10.2	3.4	10.2	4.0	0.907 ^c^
Alcohol (g/d)	35.9	28.7	32.6	30.4	0.448 ^c^
Calcium (mg/d)	406	166	407	200	0.950 ^c^
Magnesium (mg/d)	251	69	256	84	0.644 ^c^
Sodium (mg/d)	4028	1361	3950	1523	0.714 ^c^
Potassium (mg/d)	2134	655	2231	800	0.366 ^c^
Fruit (g/d)	54.1	57.9	78.1	95.2	0.039 ^c^
Vegetables (g/d)	156.2	86.4	172.0	97.3	0.240 ^c^
Physical activity (h/weekday)					
Intermediate activities	2.30	2.06	2.50	1.90	0.504 ^c^
Doing sports	0.65	0.61	0.66	0.46	0.881 ^c^
Muscle training	0.08	0.17	0.07	0.17	0.678 ^c^
Urinary excretion					
Urinary volume (mL/d)	1692	726	1701	601	0.924 ^c^
Sodium (mg/d)	4776	1728	5035	1686	0.299 ^c^
Potassium (mg/d)	1787	587	1898	582	0.195 ^c^
Sodium-to-potassium excretion ratio	2.8	1.2	2.8	1.1	0.926 ^c^

Values are presented as mean ± SD or n (%). ^a^ Overweight was defined as a BMI ≥ 25 and <30, while obesity was defined as a BMI ≥ 30. ^b^ Data of self-reported dietary intake were obtained using a self-administered diet history questionnaire (DHQ). * *p*-values for comparison between groups “^c^” were analyzed using the *t*-test, while “^d^” were analyzed using the chi-square test. Fisher’s exact test was used whenever the expected cell frequencies were ≤5. Abbreviations: BMI, body mass index; SBP, systolic blood pressure; DBP, diastolic blood pressure; SD, standard deviation.

**Table 3:** Changes in self-reported dietary intake and lifestyle characteristics and 24 h urinary excretion of sodium and potassium during the intervention period.

	Intervention Group (*n* = 93)							Control Group (*n* = 94)							Adjusted Difference		
	Baseline		Endline		Change from Baseline			Baseline		Endline		Change from Baseline			(Intervention vs. Control)		
	Mean	SD	Mean	SD	Mean	(95% CI)	*p*-Value ^b^	Mean	SD	Mean	SD	Mean	(95% CI)	*p*-Value ^b^	Mean	(95% CI)	*p*-Value ^c^
Self-reported dietary intake ^a^																	
Energy (kcal/d)	2203	555	2122	496	−81	(−172, 9)	0.077	2224	630	2195	632	−28	(−100, 44)	0.435	−59	(−162, 44)	0.263
Protein (g/d)	65.8	18.8	64.7	18.3	−1.1	(−4.4, 2.2)	0.516	67.9	22.7	68.8	24.2	0.9	(−2.5, 4.3)	0.595	−2.6	(−7.0, 1.8)	0.250
Fat (g/d)	58.9	22.3	54.3	20.6	−4.6	(−8.2, −1.0)	0.013	64.0	33.5	59.9	34.2	−4.1	(−8.3, 0.1)	0.057	−1.9	(−7.0, 3.2)	0.462
n-3 polyunsaturated fat (g/d)	2.50	0.98	2.44	0.98	−0.06	(−0.22, 0.09)	0.414	2.86	1.75	2.79	1.77	−0.07	(−0.34, 0.21)	0.625	−0.10	(−0.39, 0.20)	0.523
Carbohydrates (g/d)	279.8	87.3	270.5	68.5	−9.3	(−22.8, 4.2)	0.176	277.5	69.4	277.8	64.1	0.3	(−8.4, 9.1)	0.940	−13.9	(−58.2, 30.3)	0.535
Dietary fiber (g/d)	10.2	3.4	9.5	4.0	−0.8	(−1.5, 0.1)	0.032	10.2	4.0	10.1	4.0	−0.1	(−0.6, 0.5)	0.805	−0.6	(−1.5, 0.2)	0.110
Alcohol (g/d)	35.9	28.7	36.3	29.3	0.4	(−3.2, 4.0)	0.823	32.6	30.4	33.1	28.5	0.5	(−2.7, 3.6)	0.774	0.5	(−0.4, 5.1)	0.815
Calcium (mg/d)	406	166	369	150	−37	(−59, −16)	<0.001	407	200	397	177	−10	(−30, 9)	0.296	−27	(−52, −2)	0.038
Magnesium (mg/d)	251	69	242	75	−9	(−23, 4)	0.170	256	84	255	82	−1	(−11, 9)	0.853	−10	(−26, 6)	0.221
Sodium (mg/d)	4028	1361	3777	1302	−251.0	(−553, 51)	0.103	3950	1523	4010	1730	60.0	(−206, 326)	0.656	−278	(−634, 78)	0.125
Potassium (mg/d)	2134	655	2077	705	−57	(−173, 59)	0.331	2231	800	2217	812	−14	(−116, 88)	0.792	−65	(−212, 81)	0.382
Fruit (g/d)	54.1	57.9	63.8	71.8	9.6	(−3.4, 22.6)	0.145	78.1	95.2	76.4	80.6	−1.7	(−18.3, 14.9)	0.837	0.4	(−18.1, 18.9)	0.966
Vegetables (g/d)	156.2	86.4	140.6	112.0	−15.5	(−34.5, 3.4)	0.107	172.0	97.3	161.0	94.7	−11.1	(−26.6, 4.5)	0.160	−9.0	(−32.2, 14.3)	0.448
Lifestyle characteristics																	
Body weight (kg)	71.1	9.3	70.8	9.3	−0.3	(−0.6, −0.1)	0.005	72.7	11.1	72.5	11.1	−0.2	(−0.4, 0.0)	0.079	−0.2	(−0.5, 0.1)	0.241
Physical activities (h/weekday)																	
Intermediate activities	2.30	2.06	2.88	2.44	0.57	(0.11, 1.04)	0.016	2.50	1.90	2.42	2.05	−0.08	(−0.49, 0.33)	0.697	0.57	(0.00, 1.13)	0.049
Doing sports	0.65	0.61	1.04	3.13	0.40	(−0.22, 1.02)	0.207	0.66	0.46	0.66	0.55	0.00	(−0.09, 0.08)	0.974	0.40	(−0.22, 1.02)	0.202
Muscle training	0.08	0.17	0.11	0.23	0.03	(−0.00, 0.07)	0.051	0.07	0.17	0.08	0.19	0.01	(−0.03, 0.06)	0.511	0.02	(−0.03, 0.08)	0.348
Urinary excretion																	
Urinary volume (mL/d)	1692	726	1423	672	−268	(−391, −147)	<0.001	1701	601	1497	676	−204	(−326, −82)	0.001	−69	(−224, 86)	0.383
Sodium (mg/d)	4776	1728	4484	2019	−291	(−684, 101)	0.144	5035	1686	4784	1783	−251	(−604, 102)	0.161	−156	(−635, 324)	0.522
Potassium (mg/d)	1787	587	2222	804	435	(291, 577)	<0.001	1898	582	1640	528	−258	(−375, −140)	<0.001	646	(477, 815)	<0.001
Na-to-K excretion ratio	2.8	1.2	2.1	0.9	−0.7	(−0.9, −0.5)	<0.001	2.8	1.1	3.1	1.3	0.3	(−0.0, 0.5)	0.06	−1.0	(−1.3, −0.7)	<0.001

^a^ Data on self-reported dietary intake were obtained using a self-administered Diet History Questionnaire (DHQ). The results show a comparison of daily dietary intake, with no consideration of type of boxed lunch or miso soup. ^b^ *p*-value for paired *t*-test between baseline and endline. ^c^ *p*-value for ANCOVA adjusted for baseline values. Abbreviations: CI, confidence interval; SD, standard deviation.

**Table 4:** Changes in blood pressure during the intervention period.

	N	Baseline		Endline		Change from Baseline			Adjusted Difference (Intervention vs. Control)								
									Model I			Model II			Model III		
		Mean	SD	Mean	SD	Mean	(95% CI)	*p*-Value	Mean	(95% CI)	*p*-Value ^a^	Mean	(95% CI)	*p*-Value ^b^	Mean	(95% CI)	*p*-Value ^c^
SBP (mmHg)																	
Intervention group	93	128.7	15.7	128.3	15.0	−0.5	(−1.7, 0.8)	0.472	−2.1	(−3.6, −0.6)	0.007	−2.0	(−3.6, −0.5)	0.008	−2.0	(−3.6, −0.4)	0.011
Control group	94	127.3	12.6	129.0	13.0	1.7	(0.8, 2.6)	<0.001									
*p*-value ^d^		0.485		0.724													
DBP (mmHg)																	
Intervention group	93	83.5	10.4	83.0	10.1	−0.4	(−1.2, 0.4)	0.270	−0.9	(−2.0, 0.2)	0.116	−0.9	(−2.0, 0.3)	0.107	−0.9	(−2.1, 0.3)	0.115
Control group	94	82.7	10.1	83.2	10.5	0.5	(−0.3, 1.3)	0.226									
*p*-value ^d^		0.617		0.902													

*p*-values are for tests comparing mean differences between baseline and endline and changes in the variables between the two groups. ^a^ Model I: *p*-value for comparison of mean differences between the two groups using the ANCOVA model after adjusting for baseline values. ^b^ Model II: *p*-value for comparison of mean differences between the two groups using the ANCOVA model, after adjusting for baseline value, age, smoking status, change in body weight, and change in physical activity. ^c^ Model III: *p*-value for comparison of mean differences between the two groups using the ANCOVA model, after adjusting for baseline value, age, smoking status, change in body weight, change in physical activity, and changes in alcohol, n-3 polyunsaturated fat, energy, dietary fiber, calcium, magnesium, and protein intake. ^d^ *p* values for comparison between groups were analyzed using the *t*-test. Abbreviations: CI, confidence interval; SBP, systolic blood pressure; DBP, diastolic blood pressure.

## Appendix A

The nutritional composition of the major new low-Na, high-K foods and regular foods used in boxed lunches in this study is shown in Table A1. Note that the foods listed are those produced at the time of the intervention study (2009–2010) and differ from the currently produced versions.

**Table A1:** Nutritional composition of new low-sodium, high-potassium foods, and regular foods used in the boxed lunches in the intervention study.

Seasonings/Processed Foods	Type	Nutritional Value (per 100 g of Ingredients)						Change Rate Compared to Regular Ingredients ^a^	
		Energy	Protein	Fat	Carbohydrates	Sodium	Potassium	Sodium	Potassium
		(kcal)	(g)	(g)	(g)	(mg)	(mg)	(%)	(%)
Salt	Regular	0	0.0	0.0	0.0	39,000	100		
	New	4	0.7	0.0	0.3	23,334	20,672	−40.2	20,572.0
Soy sauce	Regular	66	8.5	0.4	7.7	5570	478		
	New	70	8.6	0.1	8.6	3330	4460	−40.2	833.1
Miso	Regular	163	12.8	5.8	14.8	3955	474		
	New	182	13.3	5.2	20.4	3028	2934	−23.4	519.0
Seasoning soy sauce	Regular	187	4.3	0.1	42.2	4170	200		
	New	72	4.2	0.1	13.5	2800	1840	−32.9	820.0
Japanese stock powder	Regular	242	27.0	0.0	33.0	13,700	180		
	New	311	34.0	0.0	43.0	8100	294	−40.9	63.3
Chicken stock powder	Regular	184	16.0	1.5	26.4	18,920	3080		
	New	260	13.4	1.4	49.6	11,200	2980	−40.8	−3.2
Seasoning mix for rice, shiso	Regular	209	8.7	1.3	40.6	17,700	738		
	New	201	8.8	2.3	36.3	11,000	8900	−37.9	1106.0
Seasoning mix for rice, wakame	Regular	184	15.4	2.2	32.0	18,000	56		
	New	183	14.4	1.8	34.8	11,000	7900	−38.9	14,007.1
Seasoned bonito flakes	Regular	265	28.2	3.6	29.9	2670	400		
	New	244	32.2	4.8	17.9	1920	2020	−28.1	405.0
Seasoned white sesame seeds	Regular	493	17.7	37.5	29.5	3995	284		
	New	516	18.4	38.8	27.4	2500	2300	−37.4	709.9
Seasoned black sesame seeds	Regular	509	18.2	39.6	28.5	3442	300		
	New	535	18.7	42.2	26.1	2200	1900	−36.1	533.3
Pork and chicken sausage	Regular	274	14.9	21.8	2.5	722	312		
	New	264	14.8	21.1	3.8	445	490	−38.4	57.1
Teriyaki chicken	Regular	170	19.9	8.0	4.7	950	25		
	New	173	19.8	8.3	4.8	588	350	−38.1	1300.0
Roast pork	Regular	256	17.3	20.6	0.4	730	263		
	New	234	17.6	16.7	3.2	593	610	−18.8	131.9
Hamburger steak	Regular	213	17.7	12.0	8.5	505	501		
	New	217	16.9	12.9	8.3	361	758	−28.5	51.3
Chicken meatball	Regular	144	17.9	3.9	9.3	575	222		
	New	147	17.2	4.5	9.4	384	424	−33.2	91.0
Salted salmon	Regular	238	21.0	14.8	0.5	1250	195		
	New	238	21.0	14.8	0.5	580	965	−53.6	394.9
Roasted salted roe with red hot pepper powder	Regular	125	24.0	1.4	4.1	2000	110		
	New	112	20.8	1.2	4.4	1000	1100	−50.0	900.0
Imitation crab meat made from surimi	Regular	98	9.0	2.0	11.0	870	8		
	New	90	10.4	0.8	10.4	450	540	−48.3	6650.0
Fried surimi	Regular	132	9.6	3.5	15.6	890	27		
	New	132	9.9	3.8	14.6	448	630	−49.7	2233.3
Dried plum paste	Regular	44	0.9	0.4	9.2	7880	440		
	New	44	0.9	0.4	9.2	4700	4400	−40.4	900.0
Dried plum	Regular	72	1.3	0.2	16.3	4100	130		
	New	72	1.3	0.2	16.3	2100	2230	−48.8	1615.4
Pickled cucumber in soy sauce	Regular	56	3.4	0.4	9.8	1600	64		
	New	41	3.4	0.4	7.3	1200	670	−25.0	946.9
Shibazuke: Kyoto-style pickles	Regular	44	2.3	0.3	8.0	1900	38		
	New	36	2.2	0.3	7.3	1300	620	−31.6	1531.6
Pickled radish	Regular	50	1.0	0.2	12.6	2100	8		
	New	48	1.0	0.1	12.0	1300	1200	−38.1	14,900.0
Pickled mustard leaf	Regular	29	1.6	0.7	4.1	1800	59		
	New	26	1.9	0.6	5.2	1300	770	−27.8	1205.1

The foods used in this study were developed between 2009 and 2010 and differ from the currently developed versions. ^a^ The rate of change (%) is calculated using the following formula: ((novel ingredient) − (regular ingredient))/(regular ingredient) × 100.
